# Endothelial dysfunction as a nexus for endothelial cell-cardiomyocyte miscommunication

**DOI:** 10.3389/fphys.2014.00328

**Published:** 2014-08-26

**Authors:** Thorsten M. Leucker, Steven P. Jones

**Affiliations:** ^1^Division of Cardiology, Johns Hopkins University School of MedicineBaltimore, MD, USA; ^2^Department of Medicine – Cardiovascular, Institute of Molecular Cardiology, and Diabetes and Obesity Center, School of Medicine, University of LouisvilleLouisville, KY, USA

**Keywords:** endothelial function, paracrine factors, cardiomyocyte, juxtacrine communication, cardiovascular disease

## Abstract

Most studies of the heart focus on cardiomyocytes (CM) at the exclusion of other cell types such as myocardial endothelial cells (EC). Such mono-cellular approaches propagate the presumption that EC provide a mere “passive lining” or supportive role. In fact, EC contribute to a dynamic network regulating vascular tone, cardiac development, and repair. Two distinct EC types, vascular EC and epicardial EC, possess important structural and signaling properties within both the healthy and diseased myocardium. In this review, we address EC-CM interactions in mature, healthy myocardium, followed by a discussion of diseases characterized by EC dysfunction. Finally, we consider strategies to reverse EC-CM “miscommunication” to improve patients' outcomes in various cardiovascular diseases.

The human heart consists of a plurality of cell types, with fibroblasts and other connective tissue cells being most abundant; the remaining cell mass consists of cardiomyocytes (CM), endothelial cell (EC), smooth muscle cells, mast cells, and immune-related cells. Although CM mass is approximately 25 times that of EC mass, the smaller EC outnumber CM by roughly 3:1 (Brutsaert, 2003). CM are surrounded by dense capillary network, which is critical for maintaining constant blood flow (Brutsaert et al., 1998); however, such intermingling of CM and EC also allows for cell-to-cell signaling, which may be of even higher significance during cellular stress (e.g., ischemia). Organized communication among the various components of this syncytium is critical for normal cardiac growth, contractile performance, and rhythmicity, but also for adaptive and protective mechanisms to combat against myocardial damage. Although cells other than CM and EC contribute to cardiac homeostasis, we focus presently on potential CM-EC interactions.

## EC-CM interactions in the adult heart

Cardiac EC rely on diverse routes of communication. Endocardial EC and capillary EC share an active blood-heart barrier and influence neighboring CM through juxtacrine and paracrine signaling, whereas coronary vascular EC act indirectly on CM through changes in coronary vasomotor tone and consequent alteration of blood flow (Brutsaert, 2003). Interestingly, either cell can initiate communication; CM can act as secretory cells and are the source of many paracrine signals that affect EC. Among these are endothelin-1 (ET1), fibroblast growth factors, adenosine, and heme oxygenases—which regulate vascular tone—thus coordinating myocardial metabolic requirements (Tirziu et al., 2010). Additionally, CM paracrine signaling—namely vascular endothelial growth factors—affects growth and development of coronary vessels. Myocardial ischemia and heart failure (HF) require vascular growth to match the increased energy demands (Li et al., 1996), and failure of vascular adaption leads to progressive cardiac dysfunction (Sellke et al., 1996). Likewise, EC play pivotal roles in the bidirectional interactions between these two major cell types. Because EC dysfunction, due to a multitude of systemic diseases affecting the cardiovascular system has a major impact on CM-EC interactions, it is important to discuss the impact of EC dysfunction on EC derived factors.

## Important paracrine and autocrine factors for EC-CM communication

EC act as sensors for shear stress to regulate vascular tone. Cardiac EC can regulate contractile properties of CM. Several autocrine and paracrine signaling molecules are responsible for this important physiologic mechanism.

### Nitric oxide

Nitric oxide (NO), produced from L-arginine by three different NO synthase isoenzymes, is a pivotal signaling molecule between EC and CM. Under physiologic conditions, neuronal (nNOS) and endothelial (eNOS) NO synthase produce the majority of NO. During inflammation, inducible NO-synthase (iNOS) significantly augments NO production (Andrew and Mayer, 1999). Interestingly, oxygen free radicals produced during ischemia-reperfusion limit NO bioavailability without significantly affecting NOS activity (Paolocci et al., 2001). Similar to its effects on smooth muscle, NO affects the onset of ventricular relaxation, allowing for optimization of ventricular pump function (Paulus et al., 1994). Although CM express both nNOS and eNOS, the vast majority of NO production comes from the EC, exceeding that of CM by greater than 4:1 (Godecke et al., 2001). The role of NO in healthy myocardium as well as the adaptive changes during pathology have been widely published (Jones and Bolli, 2006). Furthermore, studies in mice have provided substantial evidence that eNOS derived NO attenuates ischemia-reperfusion injury (Jones et al., 1999, 2004), and ultimately improves survival during HF (Jones et al., 2003a).

NO bioavailability is also necessary for a vast majority of cardioprotective effects and interventions. Ischemic preconditioning (Murry et al., 1986) perfectly exemplifies such an NO-dependent cardioprotective intervention (Jones and Bolli, 2006). Interestingly, several drugs used for the treatment of hypercholesterolemia (Jones et al., 2002, 2003b) or even erectile dysfunction (Salloum et al., 2003) improve NO bioavailability and are cardioprotective.

### Endothelin-1

ET-1 is a critical regulator of cardiac pathophysiology. ET-1 is a 21-amino acid peptide produced and released by CM (Suzuki et al., 1993), EC (Kedzierski and Yanagisawa, 2001), and fibroblasts (Fujisaki et al., 1995) of the heart. In addition to its role in cardiovascular development, ET-1 modulates coronary vascular tone. Moreover, ET-1 can directly modulate cardiac muscle function by acting on its receptors [Endothelin receptor type A (ET_A_)on CM and Endothelin receptor type B (ET_B_)on cardiac EC] expressed in atrial and ventricular myocardium (Rich and McLaughlin, 2003).

Acutely, ET_B_ activation results in release of additional signaling molecules, mainly NO and prostaglandin I_2_, whereas ET_A_ stimulation causes arteriolar constriction and can result in arrhythmias. The opposing effects of ET receptor stimulation may imply that a feedback mechanism exists between CM and EC for control of vasoconstriction through the ET-1 system (Baltogiannis et al., 2005). Chronically increased ET-1 production (days to weeks) results in CM growth and is associated with maladaptive hypertrophic remodeling of the heart and progression to HF (Yorikane et al., 1993). In addition, the circulating plasma level of ET-1 is positively correlated with severity of cardiac disease and thus may be a reliable prognostic indicator of future HF (Zolk et al., 2002).

### Neuregulin-1

EC are capable of secreting factors that augment CM compensatory reaction to hemodynamic stress. Neuregulins belong to a family of growth factors that act through receptor tyrosine kinases in the epidermal growth factor receptor family. Neuregulins mediate their actions through a set of ErbB tyrosine kinase receptors (ErbB2, ErbB3, ErbB4), which stimulate cellular proliferation, differentiation, and survival of cells in several tissues including the heart (Falls, 2003). In the adult heart, Neuregulin-1 (NRG-1) expression is restricted to EC adjacent to CM, whereas ErbB2 and ErbB4 are expressed on CM (Lemmens et al., 2006).

The important role of NRG-1 in the adult heart was discovered serendipitously (Slamon et al., 2001). Trastuzumab, an inhibitory antibody to ErbB2 (human epidermal growth factor receptor 2 or HER2/neu) used in the treatment of breast cancer, can induce cardiac dysfunction and HF, suggesting an important role for ErbB2 in the heart. Indeed, numerous studies have shown that ErbB2 and ErbB4 receptor signaling are essential for maintenance of myocardial function in the adult heart because CM specific deletion of functional receptors produces dilated cardiomyopathy (Crone et al., 2002). Additionally, conditional ErbB2 deletion or heterologous NRG-1 deficiency sensitizes mice to anthracycline cardiotoxicity (Liu et al., 2005). Interestingly, increasing NRG-1/ErbB4 signaling by NRG-1 injection or ErbB4 expression induces CM proliferation and may promote myocardial repair after MI (Bersell et al., 2009). These results emphasize the important role of NRG-1/ErB4 signaling in the response of the heart to injury, and the maintenance of normal myocardial structure and function.

## Impact of dysfunctional endothelium on EC-CM crosstalk in cardiovascular diseases

Pump failure leading to congestive heart failure (CHF) is the common endpoint of a spectrum of progressive cardiovascular diseases. Many compensatory mechanisms—such as myocardial dilatation and hypertrophy, as well as neurohormonal, cytokine, and endothelial activation—precede cardiac failure; however, such myocardial (and extra-cardiac) adaptations eventually progress to a maladaptive response, and ultimately to decompensation and CHF. Maladaptation manifests as hemodynamic abnormalities, neurohormonal imbalance, cytokine overexpression, and endothelial dysfunction.

Our understanding of endothelial function has slowly evolved over recent decades. Previously, endothelial dysfunction was thought to be limited to impaired endothelial NO production and bioavailability in response to physiologic stimuli, thereby resulting in impaired vasodilatation. Today, in addition to the idea of primary impaired NO signaling pathways, the diagnosis of endothelial dysfunction also takes into account dysfunction of many other autocrine and paracrine signaling pathways leading to EC-CM miscommunication. Numerous reviews have summarized our knowledge on various diseases and stressors, such as diabetes (Roberts and Porter, 2013), hyperlipidemia/atherosclerosis (Simionescu, 2007), hemodynamic stress (shear stress) (Giles et al., 2012), inflammatory cytokines (Koh et al., 2009), and ischemia/coronary artery disease (Gutierrez et al., 2013), which can alter endothelial function and thereby actively affect EC-CM communication and ultimately lead to cardiac failure (Shantsila et al., 2012). Therapeutic intervention to prevent the adverse outcomes of endothelial dysfunction and EC-CM miscommunication, ultimately preventing HF, is subject of intense clinical investigation. Accordingly, the following sections help clarify the relationships among various cardiovascular diseases, endothelial dysfunction, and the resulting adverse consequences on EC-CM communication, with emphasis on aforementioned paracrine/autocrine factors.

### Diabetes/insulin resistance

Diabetes mellitus (DM) type 2 significantly increases the risk of cardiovascular disease, even in the presence of rigorous glycemic control. Substantial clinical and experimental evidence suggest that both DM (Jansson, 2007) and insulin resistance (Versari et al., 2009) cause endothelial dysfunction, which may diminish the communication properties of the endothelium with other cell types (e.g., CM) and promote susceptibility to cardiovascular diseases.

Endothelial dysfunction is traditionally characterized as an imbalance of vasodilation factors such as NO and prostacyclin, and vasoconstricting factors including ET-1 and angiotensin-II (Harrison, 1997; Mather et al., 2004). Several disease related factors in DM type2 (i.e., insulin resistance, hyperglycemia, hypertension, dyslipidemia, abdominal obesity, and inflammation) are associated with EC dysfunction (Calles-Escandon and Cipolla, 2001); however, looking beyond the traditional picture of imbalance of vasodilation and vasoconstriction factors, there are several other functions of paracrine/autocrine factors leading to impaired EC-CM interaction.

ET-1 production is increased during hyperinsulinemia (Potenza et al., 2005) through activation of alternative signaling pathways including mitogen-activated protein kinase (MAPK) (Gogg et al., 2009). Clinical observations indicate that the plasma level of ET-1 is increased (Takahashi et al., 1990)—and pathophysiological actions of ET-1 are enhanced—in DM type2 (Jansson, 2007). In addition, the expression of vascular ET-1 and both ET_A_ and ET_B_ receptors (ET_A_ on CM and ET_B_ on cardiac EC) is increased in various experimental models of DM (Matsumoto et al., 2004).

Studies in EC specific ET-1 knockout mice showed that chronically elevated ET-1 led to DM-induced cardiac fibrosis (Widyantoro et al., 2010). In addition, co-culture experiments using human umbilical vein EC and neonatal rat CM showed that hyperglycemia increases EC-derived ET-1 and thereby induced CM hypertrophy (Majumdar et al., 2009). Thus, targeting endothelial cell-derived ET-1 might be useful in the prevention of diabetic cardiomyopathy (DCM) through re-institution of physiological EC-CM communication.

Significant changes in the signaling in the diabetic heart, including decreased EC protein expression of NRG1 in the left ventricular myocardium, have been reported. Furthermore, DM is associated with blunted mRNA expression of CM ErbB2 and ErbB4 receptors, and decreased phosphorylation (activation) of the ErbB2 and ErbB4 receptors (Gui et al., 2012). As outlined above, NRG1/ErbB signaling plays a pivotal role in maintaining normal cardiovascular function. Because disruption of NRG1/ErbB signaling leads to dilated cardiomyopathy (Crone et al., 2002), an imbalance in the EC (NRG1)-CM (ErbB2/4) signaling may contribute to DCM.

Loss of NO bioactivity secondary to endothelial dysfunction is probably one of the most important events contributing to DM type2 pathobiology (Brownlee, 2001; Du et al., 2001). One of the proposed mechanisms of how hyperglycemia and DM reduce NO bioavailability is through an increase in oxidative stress. In short, tetrahydrobiopterin (BH4), an essential co-factor for eNOS, is oxidized to enzymatically incompetent dihydrobiopterin, which competes with BH4 for eNOS binding (Du et al., 2000). Insufficient BH4 uncouples eNOS and generates superoxide, rather than NO (Vasquez-Vivar et al., 2002).

### Atherosclerosis/coronary artery disease

Endothelial dysfunction is closely related to the progression of atherosclerosis and associated risk factors, and it establishes a transitional step in the progression to adverse events throughout the natural history of coronary artery disease (CAD). Oxidative stress underlies the progression of endothelial dysfunction to atherosclerotic lesions (Sorescu et al., 2002). Studies have shown that coronary endothelial function is impaired at an early stage of atherosclerosis and is likely an early marker, yet not detected by routine angiography (Vita et al., 1990). It is therefore not surprising that in patients with either non-obstructive or established CAD, impaired coronary vascular function coincided with cardiovascular and cerebrovascular events (Targonski et al., 2003; Lerman and Zeiher, 2005).

Diminished supply of vasodilatory agents such as NO and prostacyclin represents an obvious potential mechanism of endothelial dysfunction. In addition, vasoconstrictors, such as ET-1, are increased in EC dysfunctional states. Because myocardial oxygen extraction is effectively maximal at basal conditions, any additional metabolic demand must be met by an increase in myocardial blood flow, hence vasodilation of the coronary arteries. Blunted coronary vasodilation results in inadequate blood flow, especially during high demand, such as patients with acute coronary syndromes (ACS).

ET-1 produced by ischemic CM and EC during ACS influences the myocardium; ET-1 binding to the ET_A_ receptor promotes catecholamine release from the adrenal glands (Nagayama et al., 2000) and modulates norepinephrine release in sympathetic nerve endings in the ventricular myocardium (Isaka et al., 2007), resulting in marked adrenergic activity (Yamamoto et al., 2005). In contrast, ET_B_ activation suppresses early sympathetic drive (Yamamoto et al., 2005). In addition, ET-1 contributes to ventricular arrhythmogenesis, which is thought to be related to increased activation of inositol 1,4,5-trisphosphate receptors leading to altered calcium release (Proven et al., 2006). Studies have shown that increased activation of these receptors during certain disease states, e.g., ACS, HF or mitral valve disease may contribute to increased arrhythmogenesis (Go et al., 1995).

In addition, several other mechanisms of endothelial dysfunction contributing to the pathogenesis of ACS have been proposed (Libby, 2001). Dysfunctional EC, mostly through an increase in local inflammatory status, leads to enhanced plaque vulnerability, participates in the process of plaque rupture, and favors thrombus formation (McGorisk and Treasure, 1996; Libby et al., 2002). Thus, evaluating endothelial function in ACS may be an important tool to assess cardiovascular risk of patients with non-obstructive- or established CAD. Interventions that maintain EC-CM integrity may prevent adverse effects of CAD.

### Endothelial dysfunction and the failing heart

Coronary- and peripheral endothelial dysfunction are present in both ischemic and non-ischemic HF (Treasure et al., 1990; Kubo et al., 1991; Bitar et al., 2006). Independently of the initial underlying pathology of HF, EC dysfunction plays a major role in the progression of the disease and has important prognostic value on clinical outcomes (Fischer et al., 2005; Shechter et al., 2009; De Berrazueta et al., 2010).

During HF, EC dysfunction is not isolated to coronary EC. For example in skeletal muscle, endothelial dysfunction may explain early fatigue and exercise intolerance (Lejemtel et al., 1986) and EC-mediated vasoconstriction contributes to the increased peripheral vascular resistance in chronic HF (Katz et al., 1992). In addition, dysfunctional endothelium has been observed in renal, mesenteric, and pulmonary vasculature, which is consistent with the notion that global EC dysfunction plays an important role in HF (Ben Driss et al., 2000).

Both preclinical and human studies emphasize the importance of coronary endothelial dysfunction during HF. In particular, the identification of impaired vasodilatory responses supported the notion that decreased NO impairs myocardial perfusion and indirectly contributes to the progression of HF (Treasure et al., 1990; Neglia et al., 1995). Yet, cardiac endothelial dysfunction, similar to coronary vascular endothelial dysfunction, is an early event in the progression to fulminant HF (Maccarthy and Shah, 2000). Indeed, high concentrations of neurohormones cause selective damage to cardiac EC, and depress mechanical performance of the adjacent myocardium. Moreover, secretion of traditional paracrine/autocrine factors is indispensable for EC-CM communication, and, such secretion is altered during acute, progressing, and stable HF (Yorikane et al., 1993; Crone et al., 2002). For example recent evidence has shown that activation of the β1-adrenergic- protein kinase A pathway and the ET-1-protein kinase C pathway is crucial in positively modulating full developed force-frequency response (FFR) in cardiac muscle (Shen et al., 2013), and dysregulation of FFR is a hallmark of HF (Ross, 1998). Thus, our silo-style view of vascular vs. cardiomyocyte dysfunction requires re-evaluation.

## Clinical assessment of endothelial function and impact of interventions

Endothelial vasodilator function is a surrogate for endothelial health (Behrendt and Ganz, 2002). Endothelial function plays a key role in vascular health and endothelial dysfunction is an early event in atherogenesis, making endothelial function testing, as a means for cardiovascular risk stratification, a valuable tool for clinicians (Benjamin et al., 2004). Presently, there is no test to evaluate directly the impact of EC-CM interactions on cardiovascular health. Unfortunately, the goal of developing a non-invasive and effective test for endothelial function has proven challenging (Vita and Keaney, 2002). Several investigational methods are briefly mentioned here.

High frequency ultrasonographic imaging of the brachial artery assesses endothelium-dependent flow-mediated vasodilation, and can estimate the effectiveness of various interventions (Corretti et al., 2002). A recent study used this method to test the relative effectiveness of two different endothelial-directed drugs and found that the technique was, indeed, effective (Liu et al., 2009).

Several studies have assessed the impact of exercise on endothelial function (Werner et al., 2009). Arterial-level shear stress (>15 dyne/cm^2^) at the outer edges of vessel bifurcations can stimulate the vasculature to produce factors ultimately promoting an atheroprotective gene expression profile (Malek et al., 1999). Non-invasive techniques to further assess the impact of exercise on endothelial function are intensively studied, including magnetic resonance imaging (Galizia et al., 2014).

Some have used positron emission tomography scanning to identify increased vascular inflammation as another potential non-invasive measurement of endothelial function (Kim et al., 2010). Chronic inflammation is a well-known risk factor for cardiovascular disease (Obel et al., 2007; Triant et al., 2007). Many groups investigated the potential impact of anti-inflammatory drugs (e.g., NSAIDs) on endothelial function. The salicylate, salsalate, reduces vascular inflammation, and increases brachial artery flow-mediated dilatation in overweight/obese patients in a NFκ B-dependent manner; (Pierce et al., 2009) however, concerns have been raised about NSAIDs (Nohria et al., 2014). Further studies to evaluate the safety of anti-inflammatory therapy on the cardiovascular system are needed.

## Closing remarks

Our understanding of the impact of EC-CM miscommunication on cardiovascular health is nascent. One area of continued potential growth lies in our [in]ability to assess clinically such cell-cell interactions. Current interventions target the endothelium to reverse endothelial dysfunction and limit the impact of cardiovascular risk factors. Several failed clinical studies targeting cell-cell interactions emphasize the need to understand the molecular interactions among various cells *in situ*. Thus, efforts should be directed at understanding such interactions and developing clinical tests to characterize EC-CM (et al.) communication leading to meaningful interventions to improve cardiovascular health. We predict it will.

### Conflict of interest statement

The authors declare that the research was conducted in the absence of any commercial or financial relationships that could be construed as a potential conflict of interest.
